# The Impact of Student Debt on Career Choices among Doctor of Public Health Graduates in the United States: A Descriptive Analysis

**DOI:** 10.3390/ijerph19084836

**Published:** 2022-04-15

**Authors:** Chulwoo Park, Eric Coles

**Affiliations:** 1 Department of Public Health and Recreation, San José State University, San Jose, CA 95192, USA; 2 DrPH Coalition, 93 Longwood Ave., Brookline, MA 02446, USA; president@drphcoalition.org

**Keywords:** Doctor of Public Health (DrPH), funding, finance, debt, cross-sectional survey

## Abstract

(1) Background: As gaps in the public health workforce grow in the wake of the COVID-19 pandemic, graduates of the schools of public health, especially Doctors of Public Health (DrPH), are poised to offer relief. While there are some known recruitment issues, student debt and debt impact on career choices are understudied. (2) Methods: In the present study, we perform a descriptive analysis of the potential impact of student debt on career choices among DrPH students and alumni in the United States using a cross-sectional national online survey. A total of 203 participants (66: alumni and 137: current students) completed the survey. Descriptive statistics, a chi-squared test of independence, and content analysis were used to analyze the funding situation and its impact on career choices. (3) Results: We found that (1) 72% of current DrPH students have zero funding support for their degree, (2) scholarship opportunities for a DrPH degree are limited, especially when compared to PhD programs, and (3) student debt impacts 59% of DrPH students’ and 29% of DrPH graduates’ career choices (about 49% of all respondents). (4) Conclusions: Student debt and a misunderstanding of DrPH are likely impediments to DrPH graduates participating in the public health workforce.

## 1. Introduction

The public health workforce is critical to maintaining and developing the public health system and its infrastructure. Especially during the COVID-19 pandemic, specialized and diverse public health professionals are needed. However, according to CDC Director Dr. Rochelle Walensky, there is a shortage of 80,000 professionals across the nation to control new variants of COVID-19 [1]. An immediate cause for this shortage has been exacerbated by the wave of resignations and retirements due to untenable working conditions during the COVID-19 pandemic, with many officials facing burnout, verbal abuse, and physical threats [2,3,4,5]. Many of the state and local public health leaders either resigned, retired, or were fired due to political backlash and pandemic pressure, leading to millions of Americans living in a community that lost its public health department leaders [3]. These acute challenges produced by public health workforce shortages were inevitable. Prior to COVID-19, the shortage of public health workers had already been predicted. For example, a policy brief by the Association of Schools of Public Health (ASPH) stated, in 2008, that the public health workforce would have a shortage of 250,000 professionals by 2020 [6]. Nine years later, a 2017 national survey of public health workers found that an estimated half of the public health workforce plan to leave their organization in the next five years [7]. However, the impending public health workforce shortage was not resolved before COVID-19 hit the country. As a result, the pandemic only exacerbated the long-standing shortage of the US public health workforce.

Graduates of the schools of public health, an obvious choice, appear available to compensate for at least some of the staffing shortages. Firstly, there is a number supply of graduates. Between 1992 and 2016, over 170,000 public health degrees were conferred by US-based institutions. The number of graduates each year is also growing, with a 300% increase in annual public health graduates over the 24-year period [8]. In 2016, the last year available, more than 19,000 public health degrees, of which around 91% were at the master’s level and 9% at the doctoral level, were conferred across more than 300 institutions [8]. While these graduates could support the US public health workforce, many studies noted recruitment challenges. There are practical issues, such as a recent qualitative study that reported that public health students experienced difficulties finding public health department jobs [9]. Another impediment was the protracted delays in hiring graduates for government sector public health departments, compared to hiring timelines in other sectors [10]. Furthermore, Yeager et al. [11] identified more fundamental issues that exist, such as the need for more competitive benefits. The prospect of salary is especially prominent as a benefit for recruitment. According to an analysis of the graduates of the Columbia Mailman School of Public Health, the salaries of the government sector, non-profit, and NGOs were less than those for healthcare, consulting, and health insurance [12]. There is also evidence that the problem is exacerbated for doctorates, especially in the field of public health. Data from the Congressional Budget Office states that the average compensation of federal workers was lower than private-sector workers at the professional degree or doctorate levels [13]. According to Sellers et al., the salaries of employees with a public health doctoral degree are paid less than employees with other doctoral degrees in the governmental public health system [14]. These issues can be summarized by a statement from an editorial in 2016 for the Association of Schools and Programs of Public Health (ASPPH): “public health agencies do not know how best to recruit qualified public health workers into the vacancies [10]”.

One issue that is absent from the discussions on public health workforce recruitment is student debt, which has been identified as having an impact on career choices in other fields. Student debt is significant in public health-related fields, according to a study on Doctor of Medicine, physical therapy, and pharmacy [15]. This type of debt has been shown to impact career choices in similar fields, notably in dentistry, according to a study by Nasseh and Vujicic [16]. The issue has been extensively studied more broadly, with only a few papers noted in the present study. A study of a highly selective undergraduate institution found that debt increased the decisions made by graduates concerning higher-salary jobs and reduced the chance of graduates choosing “public interest” jobs [17]. According to a study of a nationally representative sample, the labor market outcomes were different between different classes of student-debt holders [18].

The importance of student debt on career choices in public health is very timely, given the upcoming policy and funding decisions. On 13 May 2021, the Biden–Harris Administration announced its intention to invest USD 7.4 billion in recruiting and building a new public health workforce. While the majority of the money is for the COVID-19 response (USD 4.4 billion), USD 3 billion was intended to prepare the workforce for future outbreaks and challenges [19]. However, it is unclear how this money will be used, especially if any of it will be allocated for loan repayments or forgiveness programs. A bill that is currently on the floor of the Senate represents a glimmer of hope, which would expand a loan repayment program to students with a public health degree for the first time [20]. As others have noted, the COVID-19 pandemic has presented an opportunity to significantly advance the public health workforce [21]. Policymakers need to understand the impact of student debt to decide how best to allocate the funds to improve recruitment in public health departments.

In the present study, we analyze the impact of debt on the career choices of Doctor of Public Health (DrPH) students and alumni (DrPHers), who could potentially serve as a new generation of public health leaders. DrPHers could be a key resource in replenishing the workforce. An estimated 15% of the doctoral graduates between 1992 and 2016 were DrPHers [8]. They are trained to be future transformative leaders of public health who have strong leadership skills to develop the health of the population in all aspects of society, including local, state, and federal health departments; academia; non-profit organizations; community-based organizations; and international agencies [22]. In light of the recent wave of senior-level leadership changes, public health agencies could benefit from recruiting more DrPHers. This study aims to understand (1) the current situation of funding and scholarship for DrPH programs, (2) the amount of student debt for the DrPH education, and (3) the association between student debt and career choices among DrPH alumni and students in the United States.

## 2. Materials and Methods

### 2.1. Study Design

We conducted a descriptive study using a national cross-sectional online survey titled “Doctor of Public Health (DrPH) Funding Survey 2021” for DrPHers, to investigate their financial challenges and needs during their DrPH study. We gathered quantitative and qualitative data from a cross-sectional survey of a convenience sample of DrPH students and alumni. We created descriptive statistics to describe their financial situation and the evidence of its relationship with career choices. The survey was created in Google Forms and consisted of 1 closed-answer question, 14 multiple-choice questions, and 3 optional open-ended questions for either current students or the alumni of DrPH programs in the US. The purpose of the optional short-answer questions was to add qualitative data, such as specific examples, to any quantitative results. The survey was opened on 17 September 2021 and closed on 24 October 2021. In addition, we conducted a graduate debt comparative analysis between a DrPH degree and Life Science doctorate degrees. The information for the Life Science category was collected from the publicly available Survey of Earned Doctorates (SED) dataset, which is an annual survey presented to all graduates of accredited universities within the United States, sponsored by the National Center for Science and Engineering Statistics (NCSES) within the National Science Foundation (NSF) [23]. This survey asks questions on educational history, including financial support and debt accumulation, demographic characteristics, and post-graduation plans. The latest available SED is from the year 2020.

We designed the survey to pursue the best practices by using the following strategies. First, we introduced a clear, attainable goal—the influence of student debt on career choices—for our study in the consent notice to participants at the beginning of the online survey. Second, to prevent any potential biases arising from the authors’ affiliations with a DrPH degree, the survey avoided asking leading questions and forced-choice questions to ensure the objectivity of the study. Instead, many of the multiple-choice questions included “Other” with entering text as the last option, and/or enabled respondents to choose more than one answer. Lastly, the survey was concise and clearly designed for respondents to fill out within a short period of time (expected time: less than 5 min), and all open-ended questions were optional to answer. This study received exempt registration from the Institutional Review Board (IRB) at San José State University (IRB protocol tracking number: 21181). A waiver of signed consent from the participants was approved by the IRB.

### 2.2. Participation and Eligibility

We used convenience sampling to distribute the survey using three approaches. First, we emailed the survey link, including reminders, to the DrPH Coalition’s Google Groups, which consisted of a total of 525 members as of 11 November 2021, including 136 DrPH Coalition student members; 27 early career alumni; 48 regular alumni; 22 allies (e.g., prospective students and DrPH directors who did not earn a DrPH degree); and anyone else who showed an interest in a DrPH program. Both authors were members of the DrPH Coalition at this time (CP Educational Policy Committee Chair, EC President) [24]. Second, invitations that included reminders were posted in the DrPH Coalition’s DrPH Professional and Student Group on LinkedIn, consisting of a total of 378 members as of 11 November 2021 [25]. Lastly, we emailed 20 DrPH Program Directors in the United States who have had a close connection with the DrPH Coalition to request our invitations to be forwarded to their DrPH students and alumni.

The eligibility criteria for participation were current DrPH students or alumni in the United States and its territories. A total of 203 completed responses were gathered for analysis, consisting of 137 current students and 60 alumni.

### 2.3. Analysis

We used a descriptive analytical approach. Quantitative data were gathered from categorical questions in our survey and analyzed using RStudio version 3.62, open-source software for statistical computing and graphics (RStudio, Boston, MA, USA). To obtain more evidence for a relationship between debt and career choices, we ran a chi-squared test of the debt categories with an impact on career choices. In addition, we conducted a qualitative content analysis using the optional open-ended questions using an exported Microsoft Excel sheet from our Google Forms. The codes were created as the themes emerged. The qualitative data aimed to provide and describe an additional context to the quantitative results.

## 3. Results

### 3.1. Quantitative Findings

We analyzed the baseline characteristics of the participants, DrPH degree financing information, DrPH finance outcomes and impacts, and graduate debt comparisons. In addition, we conducted a chi-squared test of independence to observe if there was a relationship between student debt and career choices.

#### 3.1.1. The Baseline Characteristics

There were 203 total responses from the current students and alumni of DrPH programs. From the total, 66 were alumni and 137 were current students. Table 1 shows the age range, type of program (full or part time), and years of experience when their program began. The average age at the start of the DrPH program was nearly 35 years for the alumni, around 33 years for the current students, and a little over 34 years combined. From the alumni, exactly two-thirds were in full-time programs and one-third in part-time programs. From the current students, 58% were in full-time programs and 42% in part-time programs. Just over 30% of alumni had less than 4 years of experience before beginning their DrPH program, which was similar for the current students. Nearly 70% of both alumni and current students had more than 5 years of experience at the start of their programs. Finally, around 88% of alumni graduated in the 2010s and later, and only 12% graduated before then. The year of alumni graduation was 3% in the 1980s (2), 9% in the 2000s (6), 55% in the 2010s (36), and 33.3% in the 2020s (22).

#### 3.1.2. Financing a DrPH

Table 2 shows the responses to the financing questions of DrPH alumni and students. Around 1/2 of the alumni reported having no funding for their DrPH degree, while more than 70% of current students reported 0 funding. Around 9% of alumni had full tuition and/or living expenses funded for their DrPH, while just over 5% of current students had similar funding. Nearly 30% of alumni had a portion of their tuition funded, while only about 17% of current students did.

We also asked about whether participants worked during their DrPH programs and, if so, whether the work was full or part-time and for the entirety or only a portion of their program. More than half of the total respondents reported working full time for their entire program, including 38% of alumni and 64% of current students. From the alumni, a 1/3 worked part-time for the entirety of their DrPH, while only 18% of current students work part-time for their entire program. Around 20% of the respondents worked either full time or part time for some of their DrPH programs. Out of those that work, more than half of the current students work outside of their educational institution while enrolled in the DrPH program. Only 32% of alumni worked outside of their educational institution. Around 15% of current students work inside their educational institution, while 27% of alumni did. Around 15% of current students work both inside and outside their educational institution, while 38% of alumni did. About 3% of all students were not working and none of the alumni reported not working at all while enrolled in their programs.

In regard to the loans, more than 1/2 of the current students are not taking loans out, while only 41% of alumni had 0 loans. A total of 44% of alumni and 37% of current students took out federal loans. A total of 10% of all respondents had a mix of private and public and private loans.

#### 3.1.3. DrPH Debt

DrPH alumni and current students had the following amounts of debt, or anticipated debt, as a result of their DrPH program: 39% of alumni and 34% of current students have no debts; around 3% of both have debts less than USD 5000 or between USD 5000 and USD 10,000; 9% of alumni and 7% of students have debts between USD 25,000 and USD 50,000; 15% of alumni and 27% of students have debts between USD 50,000 and USD 100,000; 12% of alumni and 8% of students have debts between USD 100,000 and USD 200,000; and about 3% of both have debts of more than USD 200,000 (Table 3).

**Figure 1:**
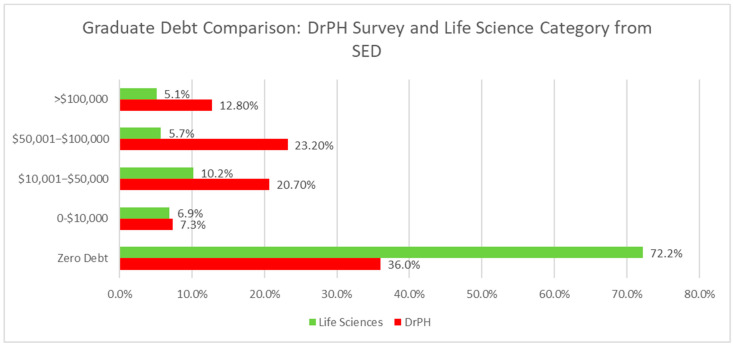
The debt comparison of the DrPH and Life Science categories from SED.

Since the SED collects data on all the doctoral graduates, we narrowed the focus to data from the “Life Science” category, which includes all “Health Sciences”, including public health, as well as agricultural sciences and natural resources, and biological and biomedical sciences. Figure 1 shows the differences between the debt of DrPH graduates and the entire Life Science category, which was the most granular data available to the public. There are noticeable differences. From the Life Science graduates in 2020, over 72% had no graduate school debts, while only 36% of DrPH students had no debts. Between USD 0 and USD 10,000 of debts, the proportions were similar, with 6.9% of SED Life Science graduates and 7.3% of DrPH grads. All other comparable debt categories presented significantly higher proportions of DrPH graduates: between USD 10,001–USD 50,000, 10.2% of Life Science graduates and 20.7% of DrPH graduates; between USD 50,001 to USD 100,000, 5.7% of Life Science graduates and 23.2% of DrPH graduates; and greater than USD 100,000 in debt, 5.1% of Life Science graduates and 12.8% of DrPH graduates.

#### 3.1.4. The Impacts on Career Choices

To the question of whether the debt situation would (or did) influence career choices, 29% of alumni and 59% of students said “yes” and the rest said “no” (Table 3).

We further analyzed the impact of debt on career choices by tabulating the responses to this question by the amount of total debt (Table 4). We found that nearly 50% of those who answered “no” had no debt, while only 22% of the “yes” respondents were without debts. Just less than 15% of both “yes/no” groups had less than USD 25,000 worth of debts. For debts between USD 25,000 and USD 50,000, 9% of the “no” group and 18% of the “yes” group were at this level. For greater amounts of debt, the percentages were 18% of “no” and 28% of “yes” had/have between USD 50,000 and USD 100,000; 8% of “no” and 11% of “yes” had/have between USD 100,000 and USD 200,000; and 1% of “no” and 6% of “yes” had/have more than USD 200,000 worth of debts.

Finally, we performed a chi-squared test of independence to obtain more evidence for a relationship between student debt (eight categories: from USD 0 to USD 200,000+) and career choices (binary: no or yes) listed in Table 4. The results are $\chi^{2}\left(7\right)=20.46,\;\;p=0.005$, rejecting the null hypothesis that student debt and career choices are independent. Thus, the independence test highlights that there is a relationship between student debt and career choices.

### 3.2. Qualitative Findings

Our qualitative data were open-ended answers to the following questions: (1) “Please list any other comments about funding resources”, (2) “Please feel free to share your thoughts about funding for DrPH programs”, and (3) an additional unique question that applied to each group. We asked for students: “If your career choice will be influenced, what career choice would you have liked to make if you had less debt?”; and we asked for alumni: “What career choice would you have liked to make if you had less debt?”. Using an inductive approach, we categorized the results into the following three common themes.

#### 3.2.1. The Limitations of Career Choices for DrPHers Due to Debt

Several responses from both alumni and students indicated that debt was limiting their career choices, most often from working in sectors with lower wages, especially government and non-profit sectors. To the question concerning whether debt would influence their career choices, one student wrote: “Government sector. But due to debt will look into private sector”. An alumnus clarified the issue of debt related to their income: “Not so focused on salary, but on what I want I’m passionate”. Another response suggested that the accumulation of debt from all education exacerbates the problem: “I would work in small, local nonprofits but I owe more than $200K for my MPH [(Master of Public Health)] (90%) and DrPH”. On the other hand, the absence of debt was viewed positively. One alumnus said: “I made career choices based on the fact that I had no debt”, suggesting that no debt opens opportunities.

#### 3.2.2. The Lack of Recognition of the DrPH

Another theme identified in the qualitative results was a lack of recognition of the DrPH among employers and academia. Several responses emphasized that employers did not know what a DrPH was or did not understand the skills it engenders. One response was: “Many employers do not understand the skills taught in DrPH programs and how applicable it is to workplaces. Too many people assume it’s a regular PhD”. Another response stated the employers did not know the “value of [a] DrPH”.

Other responses pointed to the lack of funding as evidence that academia, as well as employers, do not know the value of a DrPH. One wrote the following: “The inadequate funding for DrPH programs perpetuates institutional devaluing of the degree itself”, and went on to write that “Universities seem to resent dedication of resources to programs like the DrPH”. Another response was more direct: “The lack of funding is atrocious”. Several responses encouraged more funding for DrPH programs, such as “We must have full funding for the program like PhD’s. It’s a highly competitive program with so few students”.

#### 3.2.3. The Barriers and Opportunities to Improve DrPH Funding

Many responses noted specific barriers and potential opportunities to improve funding. Some of the specific barriers mentioned were not qualifying for scholarships due to being a part-time student, not qualifying for department-level scholarships because their DrPH program was not in a department, paying for tuition for a practicum placement while they were already working, and additional fees that were not part of tuition packages. Another issue repeatedly mentioned was the lack of loan forgiveness programs. One alumnus wrote the following: “For example, I couldn’t participate in loan repayment working as a DrPH like my colleagues with MD/ND through Indian Health Services/Tribal Health Departments. I have the same debt as they do or very similar, yet didn’t have that option”.

Several responses suggested opportunities to increase funding. One suggestion was for DrPH programs to only accept students for slots with full funding, since seeking funding added stress to their studies. Another funding idea was to help students find part-time jobs. One alumnus wrote that students should be connected with “domestic and global organizations to provide funding for the school in return for guaranteed consultancies with DrPH students over the course of the school year”. Part-time positions were also suggested to tie into practice-based learning requirements, or internships, in DrPH programs. A third approach was for tuition reimbursement from employers. However, this option might be limited as one respondent said that employer support only covered a small percentage of the total costs. Finally, a clarification of DrPH programs was also suggested as an approach to increase funding, such as “Lack of clarity for DrPH programs also makes funding challenging”, and “A clear focus and skillset will help cast funding opportunities”.

## 4. Discussion

We synthesized these results into three key findings: (1) though great variations exists among DrPH programs, the trends suggest that many students are pursuing part-time DrPH programs, which do not provide funding for their education; (2) financing a DrPH is more challenging than other Life Science degrees due, potentially, to structural barriers; and (3) the financial and funding situation of the DrPH has negative impacts on the public health workforce.

The variation in DrPH programs has been previously noted [26,27]. Our results continue to suggest program variations: students range from 0–1 years of experience to more than 19 years, a near-even split between full- and part-time programs, and some programs offer full funding and living stipends, while a majority offer no financial support. Many DrPH programs accept a part-time student status for those with more professional experience who will continue to work full-time during their study. However, funding is not provided to part-time students because they are expected to work full time and prepare all the costs of education on their own. Although part-time students, in general, are well-experienced public health professionals with several years of professional work history, for which the admissions offices are looking, they are not funded by their DrPH programs and have encountered the double difficulty of working outside of the school as well as paying for tuition and living expenses simultaneously. A higher percentage of current students were enrolled in a part-time DrPH program, compared to alumni, which may exacerbate funding issues, because part-time students qualify for fewer funding opportunities and programs may assume that full-time workers can afford tuition.

DrPHers also face more challenges to funding as highlighted by the comparison with SED. DrPHers had higher amounts of debt at every level than the Life Science category, the comparison group. Moreover, the comparison group had twice as many graduates with no debt as DrPHers. The qualitative findings suggest that this difference is known. One respondent noted: “We may now be eligible for federal loan repayment programs. In the past, we fell through the cracks as not being clinical providers”.

In line with the research in other fields, our survey found that student debt impacts career choices for DrPH graduates and may prevent recent graduates from working in the public or non-profit sectors. This point is based on two findings from our quantitative results. First, we found that nearly 6 out of 10 current students expect their debt situation to impact their career choices. Considering that there were only 238 DrPH degrees conferred in 2019 [26], our findings suggest that at least half of graduates, or 117 people, had their career choices influenced by their debt. Second, our chi-squared test found that the connection between debt influence and the amount of debt is significant. Third, the qualitative findings also support this finding. One illustrative quote is: “Government sector. But due to debt will look into private sector”.

DrPHers are needed more than ever. There have been ongoing global efforts by G7+ and the United States to defeat the COVID-19 pandemic in 2022 and advance global health security for preventing future biological catastrophes [28]. The US COVID-19 Global Response and Recovery Framework has supported US commitments in the G7+ Plan [29]. In addition, the Biden–Harris Administration created a National Strategy for the COVID-19 Response and Pandemic Preparedness to provide an actionable plan and executive actions to beat COVID-19 [30].

However, there are no concrete strategies about how to financially support the future healthcare workforce to meet those goals. According to our findings, student debt may be a barrier to the recruitment of DrPH graduates. Creating loans, LRP, and other funding mechanisms to address student debt could improve the recruitment of DrPH graduates for a new public health workforce. A DrPH degree was established to train public health professionals who would gain a strong academic ability with valuable practical experiences [26,27]. However, contrary to the original intention, our survey result indicates that a DrPH degree seems to be in the gray area due to an ambiguous approach to both academia and practice, ultimately being regarded by the public as neither a full academic terminal degree nor a full health professional degree. There is an urgent need for DrPH programs across the country to create a clear direction for a degree’s brand positioning. Although a few LRPs have become available for DrPH alumni [31,32], continuous advocacy efforts are needed because most LRPs do not clearly indicate a DrPH degree as an eligible professional doctoral degree. For example, Health Resources and Services Administration (HRSA)’s LRPs have not yet included a DrPH degree as their eligibility [33]. NIH’s Division of Loan Repayment webpage also did not explicitly add a DrPH degree to the list of Qualifying Degrees in the General Eligibility section [34].

In addition, the lack of funding and the high prevalence of debt among DrPH students during a pandemic may point to a fundamental underlying issue: the negative perception of public health. The vast majority of health funding goes to health care, rather than public health [35]. Although there were temporary boosts in public health funding, such as in the CARES Act [36], the reality is that many DrPH students have to confront high education costs without any funding support.

### The Limitations

A number of limitations need to be noted regarding this study. First, although this study was based on a national survey, it was limited by a small sample size from DrPH Coalition members and students/alumni from DrPH programs. As noted above, students and alumni who had funding issues may have been more likely to complete the survey than those who had no issues. Thus, it is likely that these results are not generalizable to the entire DrPH programs available in the United States and its territories. Among the alumni who participated in our survey, very few of them obtained a DrPH degree before the year 2010; thus, alumni data represented recent graduates from our sample, which shows the most recent situation of the financial challenge and its influence on their career choices. In addition, while marketing materials maintained a neutral tone, we are aware that a survey regarding DrPH funding from an organization that represents DrPHers likely skewed the responses. Considering these limitations on data gathering, we therefore aimed for a descriptive study to identify the traits of DrPH funding, debt, and career choices. Moreover, all the authors possessed a DrPH degree and were members of the General Assembly at the DrPH Coalition, a grassroots organization established by DrPHers across the country in the year 2019 (CP: Chair of the Education Policy Committee; EC: President) [24,25]. This affiliation may have biased the design of the survey and the sample. Second, our survey question on career choices and debt did not explicitly state that we were referring to debt limiting career choices. It appeared that some respondents understood the question as if they had no debt, then the debt still influenced their career choices by allowing them to choose the career they wanted. In addition, it could be possible that those who were in a part-time DrPH program and working full time might not be interested in finding a new job. The survey did not ask respondents about where they were working; some respondents may have already been working in the public service sector, specifically in local or state health departments. Thus, the relationship between debt and career choices would not be applicable to respondents who were not interested in a new job or who were already in the public sector. A future survey should be more explicit with the interpretation of student debt limiting their career choices and seek a more representative sample of DrPHers. Lastly, our sample size was too small to perform any significant regression analysis. The chi-squared result in this study determined that there is a relationship between career choices and student debt, but further studies should have stronger sampling strategies to ensure adequate participation for the regression analysis.

## 5. Conclusions

Recruitment for the public health workforce, especially leadership, needs to improve, in light of the workforce shortages that previously existed and were exacerbated by the COVID-19 pandemic. DrPH graduates could help to fill this gap. We surveyed over 200 DrPH students and alumni to examine DrPH funding, debt, and career choices. First, we found a significant variation in DrPH programs. The trends suggest that many students pursue part-time study, but funding opportunities are not generally provided to them because they are implicitly required to continue their day jobs to pay for their education. Second, we found a lack of funding for DrPH students, especially in comparison to PhD programs, with several barriers, such as ineligibility to scholarships and external funding. Finally, we found issues in DrPH workforce participation, specifically due to debt, with nearly half of the responses stating that debt impacts their career choices. Debt is compounded by the misunderstanding of the degree by employers, which may limit hiring and opportunities. There is a concern for the efficiency of science spending that a DrPH degree showed significantly higher debts, when compared to the Life Science category from SED. Because there is a risk of policy failure of funding allocations, policymakers are expected to know the negative effect of DrPH students’ debts on their career choices to better allocate financial resources for the successful establishment of a future public health workforce.

Student debt is an impediment to DrPH workforce participation. If DrPHers are to participate in the replenishing of the local public health workforce better, we need a better understanding of the degree in practice and more funding should be allocated to either financial aid or loan repayment.

## Figures and Tables

**Table 1:** Baseline characteristics.

		Alumni	Student	Total
Age at the start of the DrPH program	Min	23	23	23
	Mean	33.1	34.8	34.3
	Max	58	57	58
Full- or part-time program	Full-time program	66.7% (44)	57.7% (79)	60.6% (123)
	Part-time program	33.3% (22)	42.3% (58)	39.4% (80)
Years of experience	Entry level (0–12 months)	7.6% (5)	2.9% (4)	4.4% (9)
	Early career (1–4 years)	24.2% (16)	28.5% (39)	27.1% (55)
	Midcareer (5–9 years)	50% (33)	41.6% (57)	44.3% (90)
	Experienced (10–19 years)	18.2% (12)	27% (37)	24.1% (49)

**Table 2:** DrPH degree financing.

		Alumni	Student	Total
Funding	None	48.5% (32)	72.3% (99)	64.5% (131)
	Full tuition and living expenses	4.5% (3)	2.2% (3)	3% (6)
	Full tuition	4.5% (3)	2.9% (4)	3.4% (7)
	Partial tuition	28.8% (19)	16.8% (23)	20.7% (42)
	Living expenses/stipend	0% (0)	5.8% (8)	3.9% (8)
	Not sure	13.6% (9)	0% (0)	4.4% (9)
Working while in a DrPH program	Not working	0% (0)	2.9% (4)	2% (4)
	Working full time for the entire program	37.9% (25)	64.2% (88)	55.7% (113)
	Working full time for some of the program	13.6% (9)	8.8% (12)	10.3% (21)
	Working part time for the entire program	33.3% (22)	17.5% (24)	22.7% (46)
	Working part time for some of the program	15.2% (10)	6.6% (9)	9.4% (19)
If working, where	NOT APPLICABLE	3% (2)	13.1% (18)	9.9% (20)
	Outside my educational institution	31.8% (21)	56.2% (77)	48.3% (98)
	Within my educational institution	27.3% (18)	14.6% (20)	18.7% (38)
	Both within and outside my educational institution	37.9% (25)	14.6% (20)	22.2% (45)
Loans	None	40.9% (27)	51.1% (70)	47.8% (97)
	Both federal and private	7.6% (5)	3.6% (5)	4.9% (10)
	Federal	43.9% (29)	36.5% (50)	38.9% (79)
	Private	6.1% (4)	6.6% (9)	6.4% (13)
	Other	1.5% (1)	2.1% (3)	2% (4)
Other funding	Yes	56.1% (37)	33.6% (46)	40.9% (83)
	No	42.4% (28)	66.4% (91)	58.6% (119)
	Not sure	1.5% (1)	0% (0)	0.5% (1)

**Table 3:** DrPH finance outcomes and impacts.

	Categories	Alumni	Current Student	Total
Debt	0	39.4% (26)	34.3% (47)	36% (73)
	USD 1–5000	3% (2)	3.6% (5)	3.4% (7)
	USD 5001–10,000	4.5% (3)	3.6% (5)	3.9% (8)
	USD 10,001–25,000	9.1% (6)	6.6% (9)	7.4% (15)
	USD 25,001–50,000	13.6% (9)	13.1% (18)	13.3% (27)
	USD 50,000–100,000	15.2% (10)	27% (37)	23.2% (47)
	USD 100,000–200,000	12.1% (8)	8% (11)	9.4% (19)
	USD 200,000+	3% (2)	3.6% (5)	3.4% (7)
Debt situation will (or did) influence your career choices	Yes	28.8% (19)	59.1% (81)	49.3% (100)
	No	71.2% (47)	40.9% (56)	50.7% (103)

**Table 4:** The financing differences between debt career influence.

Debt Amount	No Influence	Yes Influence	Total
USD 0	49.5% (51)	22% (22)	36% (73)
USD 1–5000	3.9% (4)	3% (3)	3.4% (7)
USD 5001–10,000	3.9% (4)	4% (4)	3.9% (8)
USD 10,001–25,000	6.8% (7)	8% (8)	7.4% (15)
USD 25,001–50,000	8.7% (9)	18% (18)	13.3% (27)
USD 50,000–100,000	18.4% (19)	28% (28)	23.2% (47)
USD 100,000–200,000	7.8% (8)	11% (11)	9.4% (19)
USD 200,000+	1% (1)	6% (6)	3.4% (7)
Total	100% (103)	100% (100)	100% (203)

## Data Availability

The data presented in this study are available on request from the corresponding author. The data are not publicly available due to privacy/ethical restrictions.
